# EWS-FLI1 and RNA helicase A interaction inhibitor YK-4-279 inhibits growth of neuroblastoma

**DOI:** 10.18632/oncotarget.21933

**Published:** 2017-10-19

**Authors:** Wenjing Sun, Yesenia Rojas, Hao Wang, Yang Yu, Yongfeng Wang, Zhenghu Chen, Kimal Rajapakshe, Xin Xu, Wei Huang, Saurabh Agarwal, Roma H. Patel, Sarah Woodfield, Yanling Zhao, Jingling Jin, Hong Zhang, Angela Major, M. John Hicks, Jason M. Shohet, Sanjeev A. Vasudevan, Cristian Coarfa, Jianhua Yang, Jed G. Nuchtern

**Affiliations:** ^1^ Pediatric Surgery Division, Michael E. Debakey Department of Surgery, Texas Children’s Hospital, Baylor College of Medicine, Houston, TX 77030, USA; ^2^ Department of Pathology, MD Anderson Cancer Center, Houston, TX 77030, USA; ^3^ Department of Pediatrics, Texas Children’s Cancer Center, Baylor College of Medicine, Houston, TX 77030, USA; ^4^ Department of Molecular and Cell Biology, Baylor College of Medicine, Houston, TX 77030, USA; ^5^ Laboratory of Medical Genetics, Harbin Medical University, Harbin, Heilongjiang 150081, China; ^6^ Department of Hepatopancreatobiliary Surgery, The Second Affiliated Hospital of Harbin Medical University, Harbin, Heilongjiang 150086, China; ^7^ Department of Pathology, Texas Children’s Cancer Center, Baylor College of Medicine, Houston, TX 77030, USA

**Keywords:** neuroblastoma, YK-4-279, chemotherapy, doxorubicin, EWSR1

## Abstract

Treatment failure in high risk neuroblastoma (NB) is largely due to the development of chemotherapy resistance. We analyzed the gene expression changes associated with exposure to chemotherapy in six high risk NB tumors with the aid of the Connectivity Map bioinformatics platform. Ten therapeutic agents were predicted to have a high probability of reversing the transcriptome changes associated with neoadjuvant chemotherapy treatment. Among these agents, initial screening showed the EWS-FLI1 and RNA helicase A interaction inhibitor YK-4-279, had obvious cytotoxic effects on NB cell lines. Using a panel of NB cell lines, including *MYCN* nonamplified (SK-N-AS, SH-SY5Y, and CHLA-255), and *MYCN* amplified (NB-19, NGP, and IMR-32) cell lines, we found that YK-4-279 had cytotoxic effects on all lines tested. In addition, YK-4-279 also inhibited cell proliferation and anchorage-independent growth and induced cell apoptosis of these cells. YK-4-279 enhanced the cytotoxic effect of doxorubicin (Dox). Moreover, YK-4-279 was able to overcome the established chemoresistance of LA-N-6 NB cells. In an orthotopic xenograft NB mouse model, YK-4-279 inhibited NB tumor growth and induced apoptosis in tumor cells through PARP and Caspase 3 cleavage *in vivo*. While EWS-FLI1 fusion protein is not frequently found in NB, using the *R2* public database of neuroblastoma outcome and gene expression, we found that high expression of EWSR1 was associated with poor patient outcome. Knockdown of EWSR1 inhibited the oncogenic potential of neuroblastoma cell lines. Taken together, our results indicate that YK-4-279 might be a promising agent for treatment of NB that merits further exploration.

## INTRODUCTION

Neuroblastoma (NB) is the most common extracranial solid tumor in childhood. More than 650 cases are diagnosed each year in North America. It causes up to 10% of childhood cancer mortality while accounting for about 5% of all pediatric cancer diagnoses. In spite of intensive multimodal therapy, the 5-year survival rate in children with high-risk disease is approximately 40% and has not improved dramatically over the past two decades [1–6]. Treatment failure in high risk NB is largely attributed to the development of chemoresistance, which ultimately leads to relapse after frontline therapy [3]. One approach to solving this problem is to directly target the cellular changes induced by exposure to chemotherapy. In the past decade, the understanding of NB and new drug discovery have advanced tremendously with the help of molecular genetic techniques like genome sequencing and bioinformatic analysis, although a detailed understanding of the pathogenesis of chemotherapy resistance in this pediatric cancer remains obscure [7, 8].

To facilitate our study, we used a bioinformatic analysis to identify novel agents capable of reversing the chemoresistant phenotype in NB tumor cells. We compared the gene expression profiles of six paired pre-and post-chemotherapy NB tumor samples from high risk patients. Using the Connectivity Map bioinformatics platform (https://www.broadinstitute.org/cmap) [9, 10], we identified 10 readily available therapeutic agents that were predicted to reverse the transcriptome changes associated with chemotherapy resistance (Table 1). Given that several of these compounds have been previously reported to be active against NB cells [11–13], we examined the cytotoxicity of the remaining drugs: fluoropyruvate, PRL-3-inhibitor-1, YK-4-279, PF-04217903, zebularine, and EX-527. Initial cell viability experiments in NB cell lines showed one of these agents, the EWS-FLI1/RNA helicase A (RHA) interaction inhibitor YK-4-279, obviously inhibited the growth of NB cells *in vitro*, while the others did not.

**Table 1:** Discovery of inhibitors and target genes from NB samples by the connectivity map bioinformatics platform

Inhibitors	Target
FK866	NMPRTase inhibitor, NAMPT
Fluoropyruvate	pyruvate dehydrogenase complex
Sirolimus (rapamycin)	mTOR
MK-1775	Wee1 inhibitor, Wee1
PRL-3-inhibitor-1	PRL-3
YK-4-279	EWS-FLI1 interaction with RHA
PF-04217903	c-Met
Zebularine	DNA methyltransferase, DNMT1
EX-527	SIRT1
Saracatinib	Src/Abl Inhibitor

YK-4-279, a small molecule, was discovered in a molecular screen based on its ability to bind to EWS-FLI1 and inhibit interaction with RHA [14]. Several studies have demonstrated the anti-tumor efficacy of YK-4-279 in Ewing’s sarcoma, ERG- and ETV1-mediated prostate cancer and EWS-FLI1-induced leukemia [14–21]. Without exception, all of these malignancies harbor EWS-FLI1 fusion genes or other so called ETS translocations. Given that neuroblastoma does not typically undergo these translocation events, this agent has not previously been considered for treatment of this malignancy.

A potential advantage of an unbiased bioinformatics platform such as the Connectivity Map is discovery of targets and pathways that have not been previously implicated in NB pathogenesis. Thus neither EWSR1 nor ETS translocations have been previously implicated in NB progression. In this paper we explore the use of YK-4-279 as a therapeutic agent for NB. Our results suggest that EWSR1, or related pathways, may be effective targets for NB treatment.

## RESULTS

### YK-4-279 inhibits the viability and anchorage-independent growth ability of NB cells

**Figure 1:**
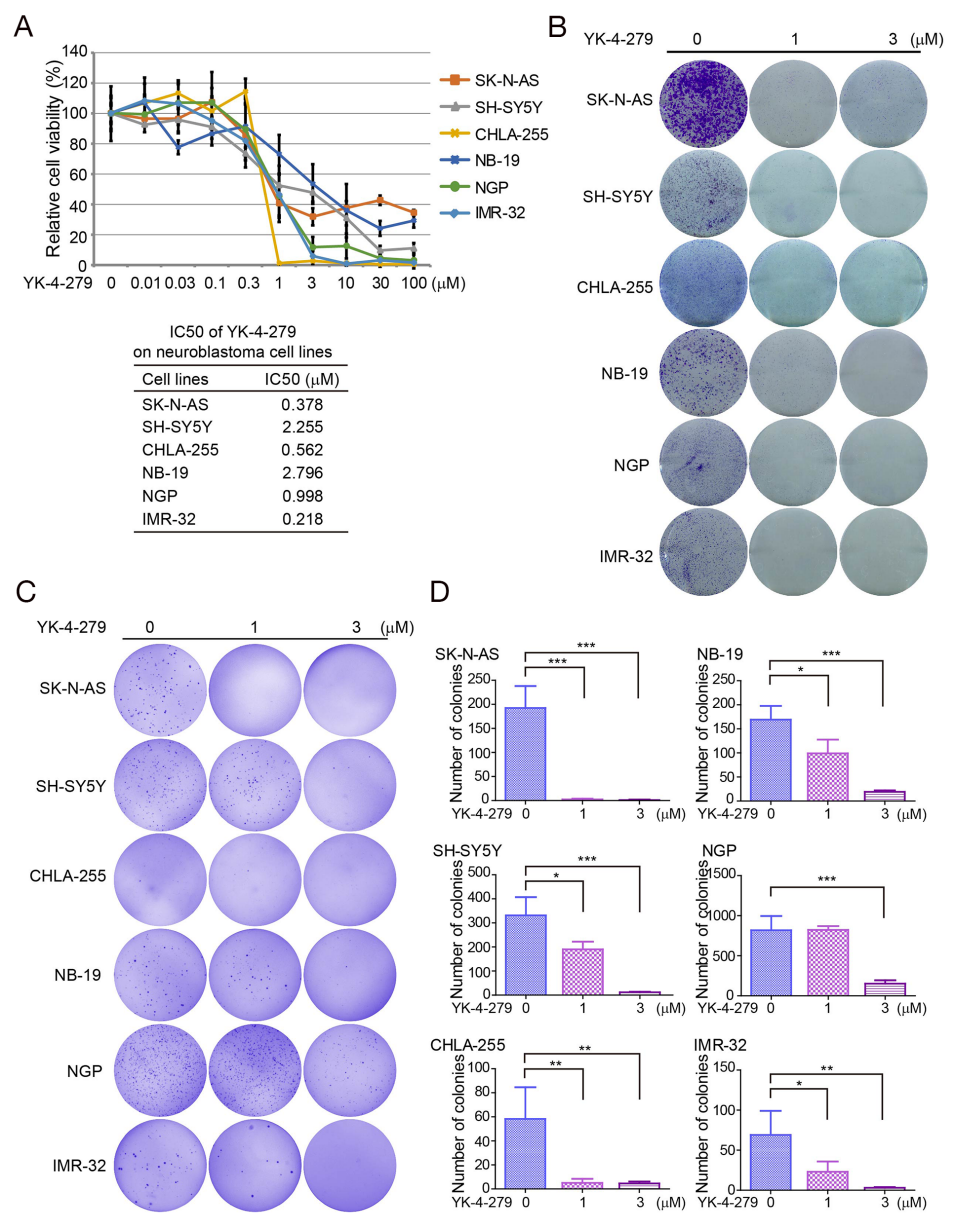
YK-4-279 inhibits the viability and anchorage-independent growth of NB cells **(A)** Cell cytotoxicity of YK-4-279 of NB cells in the CCK-8 assay. Six human NB cell lines SK-N-AS, SH-SY5Y, CHLA-255, NB-19, NGP, and IMR-32 were treated with YK-4-279 at the concentrations of 0, 0.01 μM, 0.03 μM, 0.1 μM, 0.3 μM, 1 μM, 3 μM, 10 μM, 30 μM, and 100 μM for 72 h, and then subjected to a CCK-8 assay. The absorbance of each well was measured at 450 nm and plotted for the cell viability curve. The data are represented as mean ± SD. IC50 values of YK-4-279 in NB cell lines are listed. **(B)** Colony formation of NB cells treated with YK-4-279. NB cells were seeded in 6-well plates at 5 ×10^3^ per well, and then incubated with YK-4-279 at 0, 1 μM, or 3 μM for two weeks. The colonies were fixed, stained with crystal violet, and photographed. **(C)** Anchorage-independent growth was assessed by soft agar assay. NB cells were incubated with YK-4-279 at 0, 1 μM, or 3 μM in soft agar for three weeks, followed by staining with crystal violet and photographed. **(D)** The colonies were counted and plotted. Data were represented as mean ± SD. * *P*<0.05, ** *P*<0.01, *** *P*<0.001, by *ANOVA* and Dunnett’s multiple comparison post-test.

To assess the effect of YK-4-279 on NB cells, we performed cytotoxicity assays on a panel of six NB cell lines, including three MYCN nonamplified (SK-N-AS, SH-SY5Y, and CHLA-255), and three MYCN amplified (NB-19, NGP, and IMR-32) cell lines. We found that the treatment distinctly reduced the cell viability of all types of NB cells in a dose-dependent manner (Figure 1A). The IC50 values of YK-4-279 in NB cell lines is between 0.218 μM (IMR-32) to 2.796 μM (NB-19) (Figure 1A). The cytotoxic effect of YK-4-279 was confirmed by morphological images of six NB cell lines after treatments for 72 h (Supplementary Figure 1).

To further validate the effect of YK-4-279 on growth of NB cells, the cell colony formation assay was performed. A dose-dependent inhibition of colony formation was seen in YK-4-279 treatment groups compared to the untreated cells (Figure 1B). These data demonstrate that YK-4-279 significantly suppresses cell viability and growth of NB cells, both MYCN nonamplified and amplified.

To assess whether YK-4-279 could inhibit anchorage-independent growth of NB cells, soft agar growth assays were performed with NB cell lines. In this assay, SK-N-AS, SH-SY5Y, CHLA-255, NB-19, NGP, and IMR-32 cells were cultured with YK-4-279 for three weeks. We observed that the numbers of colonies were markedly decreased in YK-4-279 treated groups compared to the control cells in all the tested cell lines (Figures 1C and 1D). The results indicate that YK-4-279 impairs anchorage-independent growth of NB cells.

### YK-4-279 induces cellular apoptosis in NB cells

**Figure 2:**
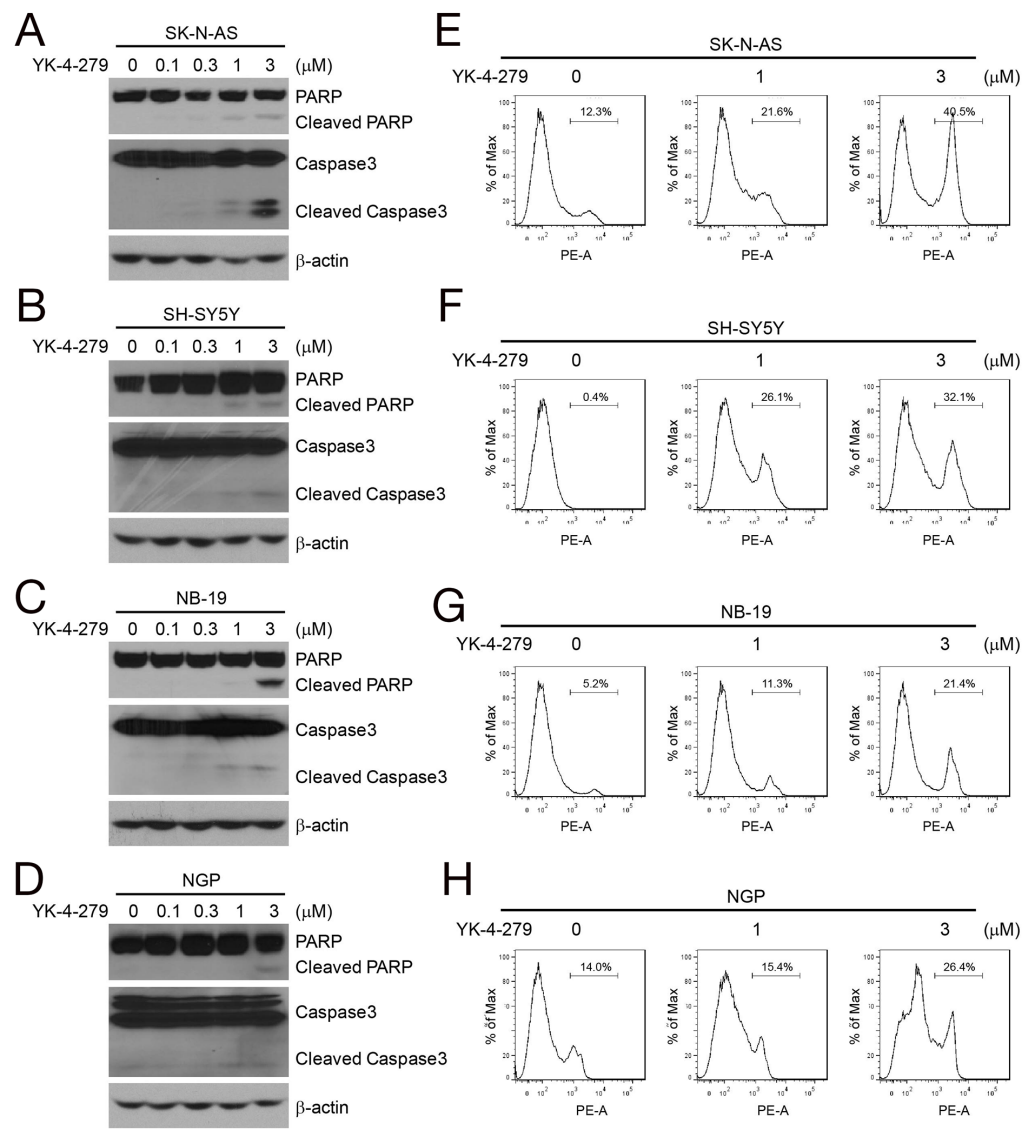
YK-4-279 induces apoptosis of NB cells **(A-D)** YK-4-279-induced cell apoptosis of NB cells by Western blot assay. NB cell lines SK-N-AS, SH-SY5Y, NB-19, and NGP were treated with YK-4-279 (0, 0.1 μM, 0.3 μM, 1 μM, 3 μM) for 24 h. Whole cell lysates were subjected to SDS-PAGE and immunoblotted with antibodies against PARP and Caspase 3 to detect apoptosis. β-actin was detected as loading control. **(E-H)** YK-4-279-induced apoptosis of NB cells by FACS. Cells were treated with YK-4-279 (0, 1 μM, 3 μM) for 24 h, and then stained by PI and analyzed by FACS.

YK-4-279 has been reported to induce apoptosis in many tumor types, including sarcoma and prostate cancer [14, 19]. We investigated whether YK-4-279 was capable of inducing apoptosis in NB cells using four NB cell lines, two *MYCN* nonamplified (SK-N-AS and SH-SY5Y), and two *MYCN* amplified (NB-19 and NGP). The cells were treated with YK-4-279 at different concentrations (0, 0.1 μM, 0.3 μM, 1 μM, 3 μM) for 24 h, and cell lysates were studied using immunoblotting for PARP, and Caspase 3. YK-4-279 induced PARP and Caspase 3 cleavage in all the tested cell lines (Figures 2A–2D). Additionally, PI staining and FACS analysis was performed to analyze the cells for apoptosis after treatment with YK-4-279. We found that the population of apoptotic cells increased with YK-4-279 treatment in a dose-dependent manner (Figures 2E–2H).

### YK-4-279 shows anti-tumor efficacy in orthotopic xenograft mouse models of NB

**Figure 3:**
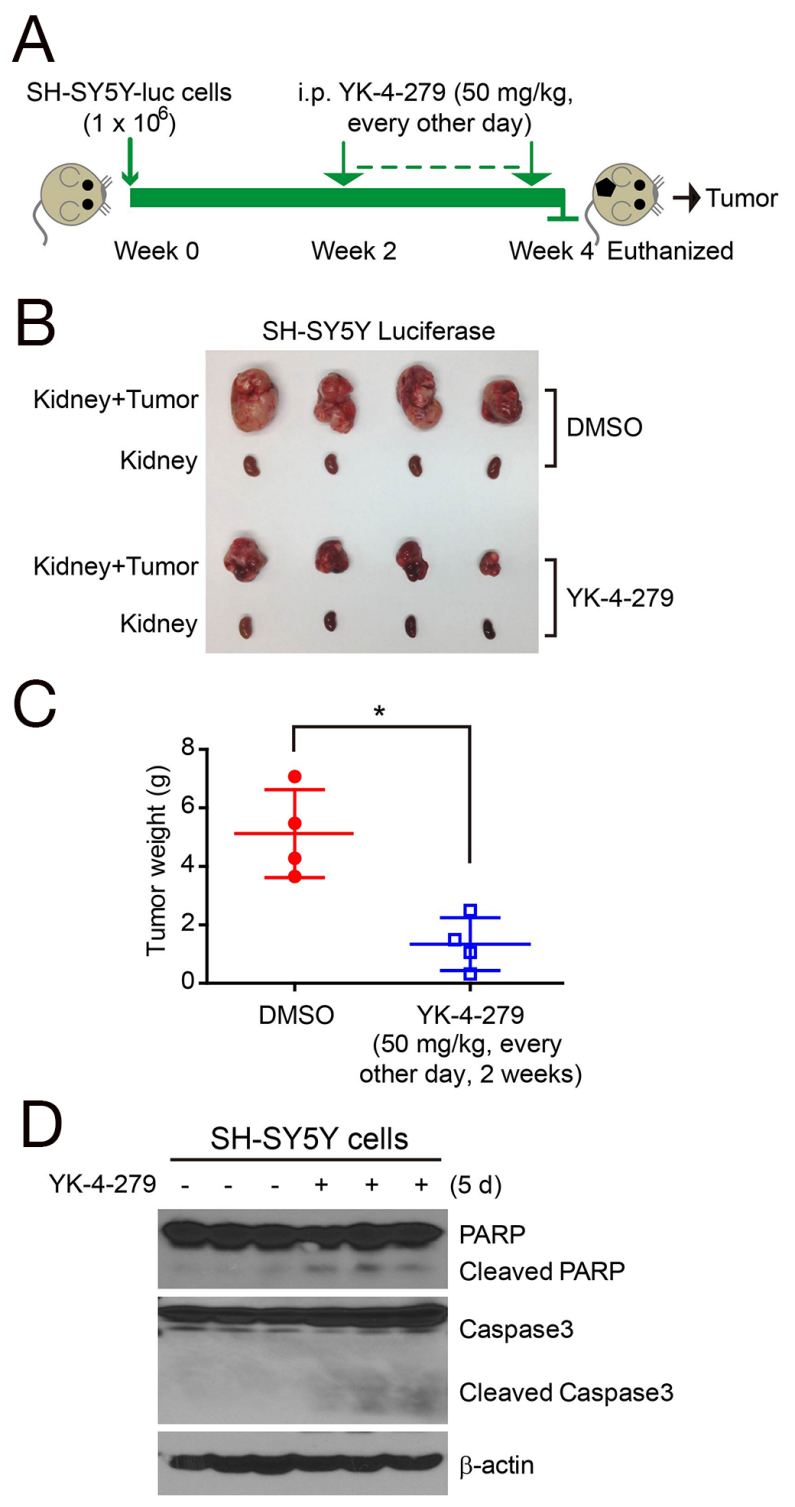
YK-4-279 inhibits tumor growth in orthotopic NB xenograft mouse models **(A)** Schematic representation of experimental plan to analyze the effect of YK-4-279 on NB *in vivo*. One million SH-SY5Y-luc cells were injected under the sub-renal capsule of mice to develop orthotopic xenografts. Implanted mice were treated with YK-4-279 (i.p. injection, 50 mg/kg, every other day) or vehicle control (DMSO) starting the day after implantation for two weeks, followed by necropsy at four weeks. **(B)** Photographs of SH-SY5Y xenografted tumors and the corresponding kidney controls from DMSO control group and YK-4-279 treatment group were taken at the end of treatment. **(C)** SH-SY5Y-derived tumor weights from control (n=4) and treatment groups (n=4) were presented as the mean with SDs. * indicates *P*<0.05 with *t*-test analysis, two-tailed. **(D)** The mice bearing SH-SY5Y xenograft tumors for four weeks were treated with DMSO or YK-4-279 by i.p. injection for five days, and then the tumors were resected and subjected to SDS-PAGE, and immunoblotted with the anti-PARP and Caspase 3 antibodies. β-actin was used as a loading control.

Based on the cytotoxic effects of YK-4-279 on NB cells *in vitro*, we proceeded to assess the drug’s effect on inhibiting tumor growth in an orthotopic xenograft mouse models of NB [22, 23]. In this set of *in vivo* experiments, SH-SY5Y cells with stable expression of the luciferase gene were implanted into the left kidneys of nude mice. Two weeks later, mice were treated with YK-4-279 or DMSO *via* i.p. injection every other day for an additional two weeks. At the end of the YK-4-279 treatment, the xenograft tumors of SH-SY5Y from control and treatment groups were dissected and weighed (Figure 3A). Significant tumor growth inhibition was observed in YK-4-279 treatment groups compared with the control groups (Figure 3B). Treatment of SH-SY5Y xenograft mice with YK-4-279 resulted in decreased tumor weight (Figure 3C). In order to test activation of apoptosis with YK-4-279 treatment *in vivo*, mice bearing SH-SY5Y tumors for four weeks were treated with either YK-4-279 or DMSO *via* i.p. injection for five days. Tumors from these mice were harvested and analyzed for activation of apoptotic pathways using immunoblotting. In this assay, YK-4-279 induced the cleavage of PARP and Caspase 3 *in vivo* (Figure 3D).

### YK-4-279 enhances the cytotoxic effect of Dox and Dox-induced apoptosis in NB cells

**Figure 4:**
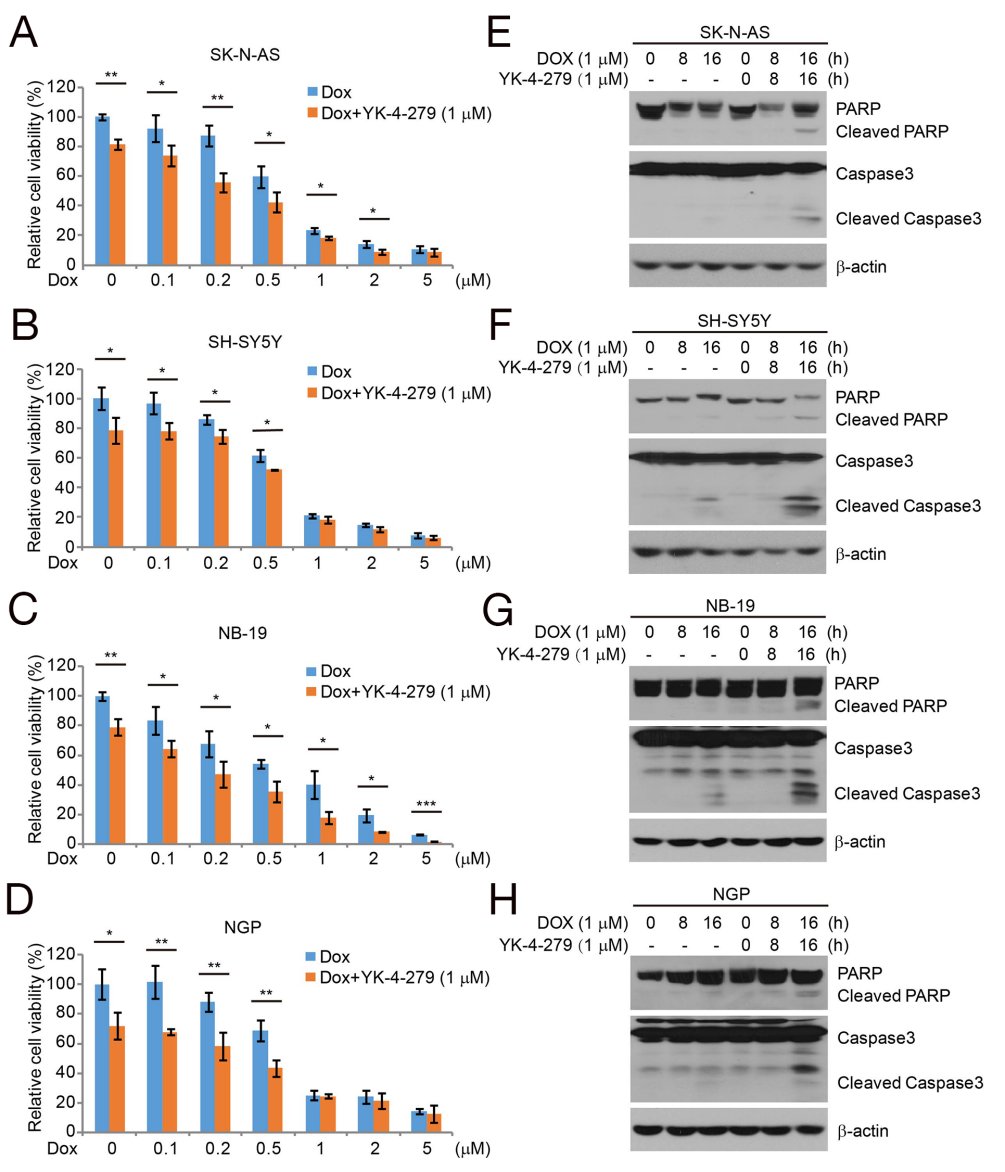
YK-4-279 enhances the cytotoxic effect of Dox and Dox-induced apoptosis in NB cells **(A-D)** The NB cell lines SK-N-AS, SH-SY5Y, NB-19, and NGP were treated with Dox at the concentrations of 0 μM, 0.1 μM, 0.2 μM, 0.5 μM, 1 μM, 2 μM, 5 μM with or without YK-4-279 1 μM for 48 h. The cell viability was then measured by CCK-8 assay. The data are represented as mean ± SD. * *P*<0.05, ** *P*<0.01, *** *P*<0.001, by *t*-test. **(E-H)** The NB cells were treated with Dox (1 μM) alone or combined with YK-4-279 (1 μM) for 0, 8 h, or 16 h, whole cell lysate were subjected to SDS-PAGE and immunoblotted with antibodies against PARP and Caspase 3 to detect the apoptosis. β-actin was used as a loading control.

Dox is an effective chemotherapeutic drug in NB cells. We evaluated whether YK-4-279 could enhance the effect of Dox on NB cell lines. Four NB cell lines, SK-N-AS and SH-SY5Y (*MYCN* nonamplified), and NB-19 and NGP (*MYCN* amplified) were cultured in increasing concentrations of Dox alone or in combination with YK-4-279 (1 μM) for 48 h, and then analyzed using the CCK-8 assay. Cell viability was much lower when the NB cells were treated with Dox combined with YK-4-279 compared to the Dox alone (Figures 4A–4D). Subsequently, we were able to show that combination treatment of NB cell lines with YK-4-279 and Dox enhanced PARP and Caspase 3 cleavage (Figures 4E–4H).

### YK-4-279 has a cytotoxic effect on chemo-resistant NB cells

**Figure 5:**
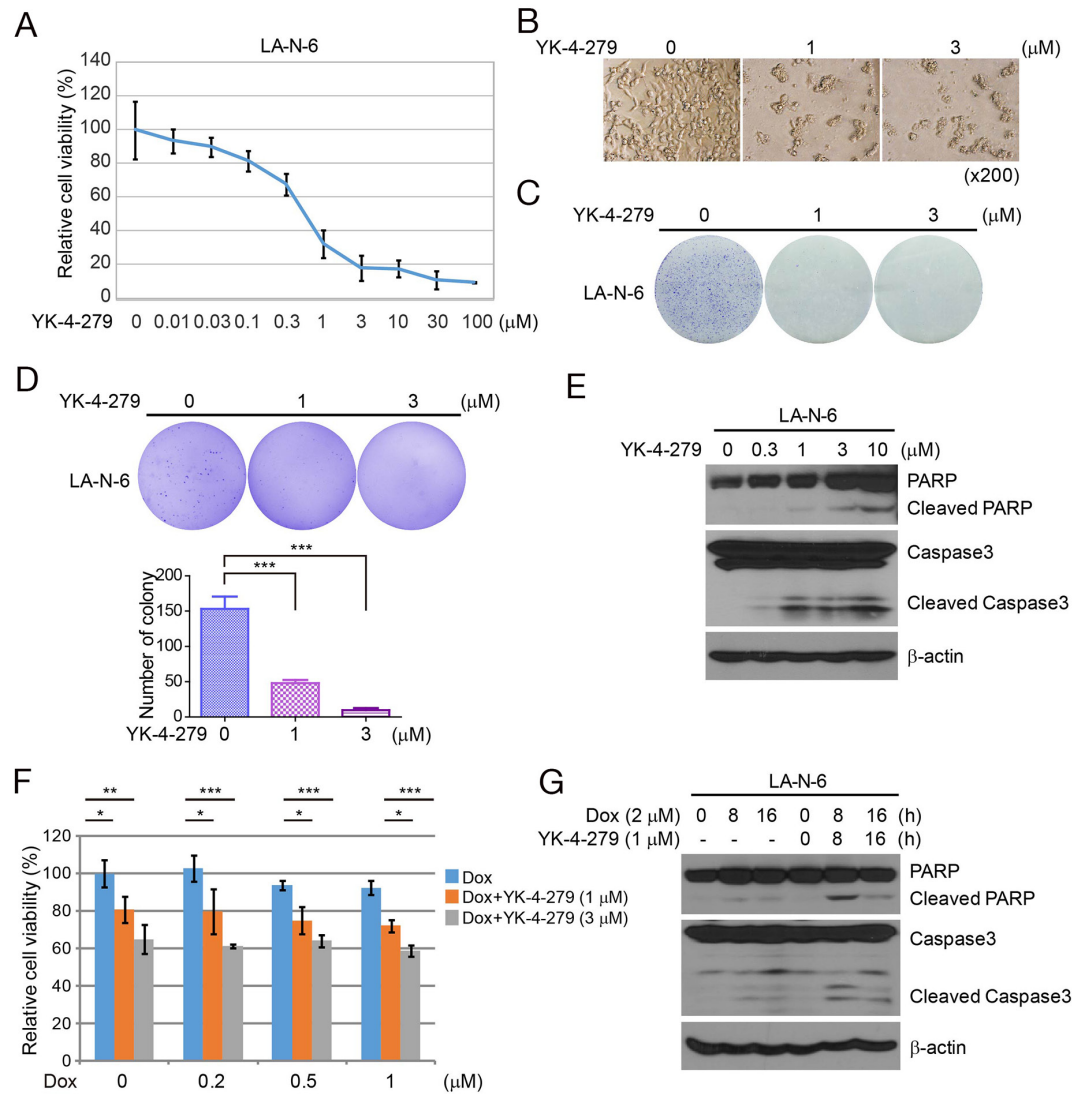
YK-4-279 has the cytotoxic effect on chemo-resistant NB cell line LA-N-6 **(A)** YK-4-279 decreases cell growth of chemo-resistant NB cell line LA-N-6 in the CCK-8 assay. LA-N-6 cells were treated with YK-4-279 at the concentrations of 0, 0.01 μM, 0.03 μM, 0.1 μM, 0.3 μM, 1 μM, 3 μM, 10 μM, 30 μM, and 100 μM for 72 h, and then subjected to the CCK-8 assay. **(B)** Photographs of cells of A. **(C)** YK-4-279 decreases colony formation of LA-N-6 cells. **(D)** YK-4-279 impairs anchorage-independent growth of LA-N-6 cells. **(E)** YK-4-279 induces PARP and Caspase 3 cleavage of LA-N-6. **(F)** YK-4-279 enhances the cytotoxic effect of Dox in LA-N-6. **(G)** YK-4-279 enhances Dox-induced PARP and Caspase 3 cleavage in LA-N-6.

LA-N-6 is a chemotherapy-resistant NB cell line. We analyzed the effect of increasing concentrations of YK-4-279 on LA-N-6 cells. We found YK-4-279 reduced the viability of LA-N-6 cells with an IC50 of 0.653 μM (Figure 5A and 5B). This effect was also confirmed by a colony formation assay in which YK-4-279 decreased colony formation by LA-N-6 cells (Figure 5C). To assess whether YK-4-279 could inhibit anchorage-independent growth of LA-N-6 cells, soft agar assays were performed and showed that the numbers of colonies were markedly decreased in YK-4-279 treated groups compared with control cells (Figure 5D). Immunoblot assays of LA-N-6 cells treated with YK-4-279 revealed cleavage of PARP and Caspase 3 in LA-N-6 cells (Figure 5E). Furthermore, we found that YK-4-279 enhanced the cytotoxic effect of Dox in LA-N-6 cells as detected by the CCK-8 assay (Figure 5F). YK-4-279 also strengthened Dox-induced PARP and Caspase 3 cleavage in these cells (Figure 5G).

### EWSR1 expression strongly correlates with mortality of NB patients and is required for NB cell growth

**Figure 6:**
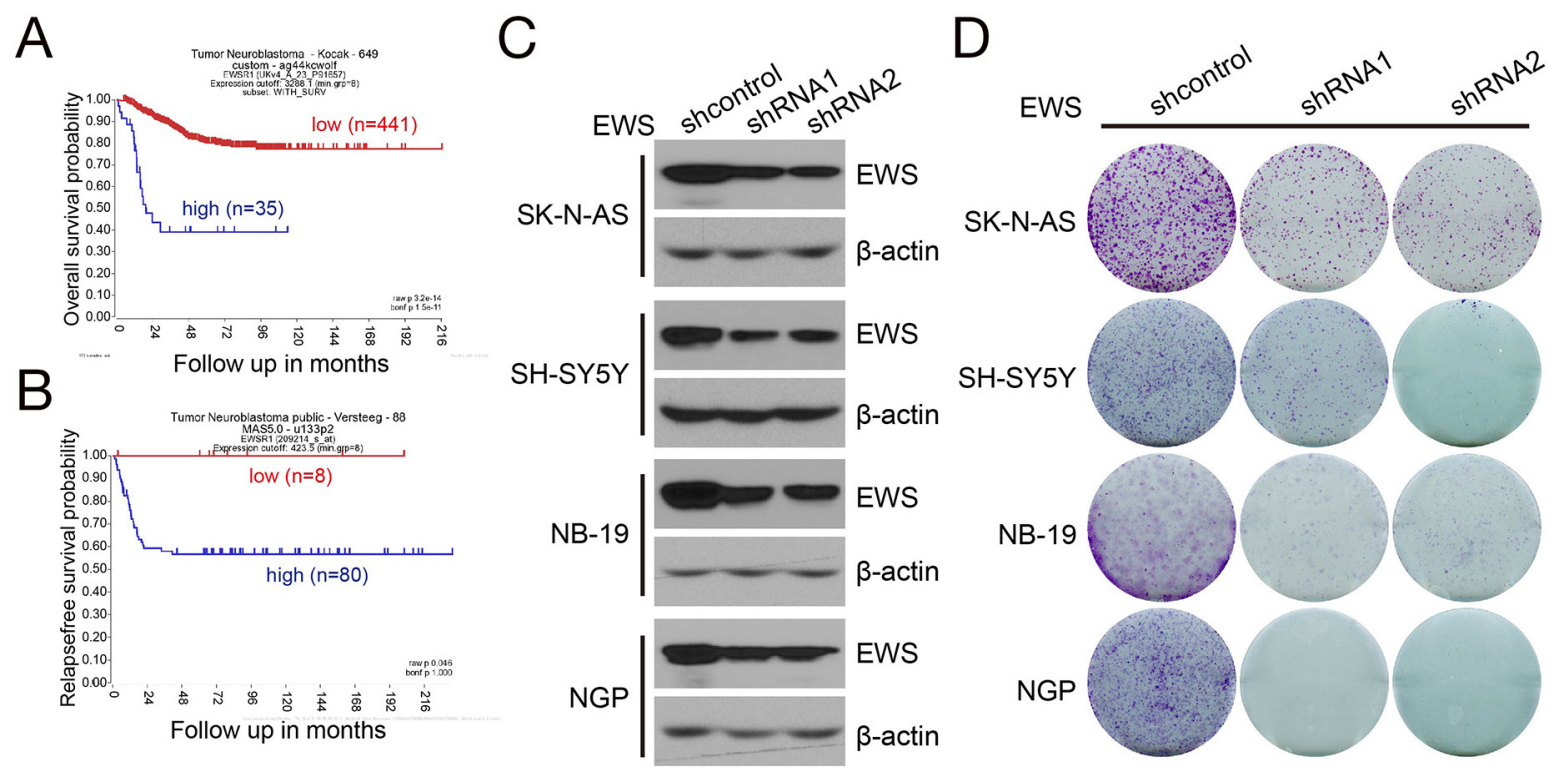
EWSR1 expression correlates with survival of NB patients and is required for NB cell growth **(A, B)** Kaplan-Meier curves shows the probability of survival of 476 patients in the Kocak dataset (A) and 88 patients in the Versteeg dataset (B) based on EWSR1 expression level. **(C)** Stable knockdown of *EWS* in NB cells. SK-N-AS, SH-SY5Y, NB-19 and NGP cells were stably transduced with lenti-shcontrol, lenti-shEWS-1 or lenti-shEWS-2 virus by puromycin selection for five days. The whole cell lysates were then subjected to SDS-PAGE and immunoblotted with antibodies against EWS. β-actin was detected as loading control. **(D)** Colony formation of *EWS* stable knockdown NB cells. *EWS* stable knockdown SK-N-AS, SH-SY5Y, NB-19 and NGP cells were seeded in 6-well plates at 5 ×10^3^ per well, and then incubated for two weeks. Cell colonies were fixed, stained with crystal violet, and photographed.

Knowing that the EWS-FLI1 fusion protein is not frequently found in NB [24, 25], we tried to evaluate the functional impacts of EWS and FLI1, respectively, in this disease. Currently, hundreds of samples of patients with NB have been examined by expression array or RNA sequencing, which provide a unique opportunity to address this issue [26, 27]. R2: Genomic Analysis and Visualization Platform (http://hgserver1.amc.nl/cgi-bin/r2/main.cgi) provides publically available gene expression databases for multiple tumors. The Kocak dataset and Versteeg dataset include NB profiles with both gene expression data and patient outcome. Since there was no data available for EWS-FLI1 in public NB datasets, we analyzed the expression of EWSR1 and FLI1, respectively, in NB. Kaplan-Meier analysis of the Kocak (n=476) and Versteeg (n=88) NB tumor gene expression datasets revealed that high EWSR1 transcript levels strongly correlate with poor survival (Figures 6A and 6B). There is no significant correlation between FLI1 expression and survival of NB patients (Supplementary Figure 2A). High expression of EWSR1 is observed in the Jagannathan 38 NB cell lines dataset (Supplementary Figure 2B), as well as in the NB cell lines used in this study (Supplementary Figure 2C).

We studied knockdown of EWSR1 expression to assess whether this perturbation impaired NB cell growth. We generated stable EWSR1 knockdown cells from the parent SK-N-AS and SH-SY5Y (*MYCN* nonamplified), and NB-19 and NGP (*MYCN* amplified) cell lines using two EWSR1 shRNAs, respectively (Figure 6C). In a cell colony formation assay, we found that knockdown of EWSR1 in NB cells inhibited cell growth (Figure 6D). These findings suggest that EWSR1 is an important factor influencing the biology and response to therapy of NB.

In further analysis, we sought to determine whether EWSR1 is directly regulated by MYCN since MYCN is a primary oncogenic driver of NB and is highly expressed in more aggressive tumors [28]. In the Kocak dataset with 649 NB samples, EWSR1 is overexpressed in both *MYCN* amplified and nonamplified samples, although the expression in the *MYCN* amplified group is higher than in the *MYCN* nonamplified group (Supplementary Figure 2D). This may explain why YK-4-279 works well in both *MYCN* amplified and nonamplified cell lines. MYCN-ChIP-seq data [29] analysis of EWSR1 revealed that MYCN binds to the 5’-UTR of EWSR1 in both MYCN high and low expression NB cells suggesting that EWSR1 may be directly regulated by the MYCN oncoprotein (Supplementary Figure 2E). Furthermore, bioinformatic investigation using the R2: Time Series dataset revealed that lentivirus-mediated knockdown of MYCN is associated with lower expression of EWSR1 in NB cells (Supplementary Figure 2F). Taken together, these results suggest that EWSR1 expression influences tumor progression in NB and is partially regulated by MYCN oncoprotein.

## DISCUSSION

We have demonstrated that the EWS-FLI1 and RHA interaction inhibitor YK-4-279 is active against neuroblastoma cell lines *in vitro* and in a mouse xenograft model of NB. This drug inhibits anchorage independent growth in these tumor cell lines and causes death by standard apoptotic pathway. The molecule is active against a chemotherapy-resistant cell line and its effect is additive with Dox.

The EWS-FLI1 or EWS-ETS fusion has been regarded as the hallmark of Ewing’s sarcoma and related tumors, and is found in nearly 95% of tumors [25, 30, 31]. The small molecule YK-4-279 was precisely designed to block the binding between EWS-FLI1 and RHA and downregulate the transcriptional activity of this oncogenic fusion protein, inhibiting the growth of Ewing’s sarcoma [14]. Since the anti-tumor efficacy of YK-4-279 in Ewing’s sarcoma was demonstrated in 2009, ERG/ETV1-mediated prostate cancer and EWS-FLI1-induced leukemia have been verified as additional target diseases [14–21]. Nonetheless, there is no definitive evidence that the presence of an ETS translocation, rather than mere overexpression of an ETS transcription factor, is necessary for YK-4-279 to be an effective agent [32]. In addition, a recent study showed that YK-4-279 treatment did not mimic the transcriptional effects of EWS-FLI1 knockdown [33]. Instead, they observed a pattern of alternative splicing events that were similar to EWS-FLI1 reduction. This study suggested that YK-4-279 disrupts the protein interactions between EWS-FLI1 a broad range of proteins involved in RNA slicing thus altering the splicing program rather than changing the gene expression pattern of susceptible cells [33]. These findings indicate the mechanism of YK-4-279 action may be more complex than direct alteration of the transcriptome.

It should be noted that our research used an unbiased bioinformatics platform to predict reagents that might reverse the chemoresistance phenotype of NB cells, rather than an inductive approach based on expression profiling that identified overexpression specific oncogene products in chemoresistant cells. This scheme led us to test an agent designed to interfere with EWS-FLI1 and RHA interaction in a tumor that has not been considered to be driven by this fusion protein. Given that EWSR1-FLI1 type fusion transcripts are not frequently found in NB [34], we investigated the role of each of the fusion partners in NB tumor progression. Using the R2 platform, we analyzed the expression level of EWSR1 and FLI1 in published NB tumor and cell line gene expression datasets. These results showed that compared to FLI1, EWSR1 is generally expressed at a higher level in NB tumors and cell lines. Furthermore, EWSR1 expression is a poor prognostic factor in NB. (Figure 6). Thus it seems likely that in NB there is either a different fusion partner for *EWS,* such as a different member of the ETS transcription factor family, or there is a direct effect of YK-4-279 on EWSR1 in these cells. The expression array data in the public domain is not sufficient to differentiate between these two possibilities and further investigation is warranted.

It is well established that *MYCN* amplification is associated with poor prognosis in NB [28, 35]. We analyzed the correlation between *MYCN* status and EWSR1 through the UCSC Genome Bioinformatics and public datasets in R2. The analysis indicated that MYCN binds to the *EWSR1* 5’-UTR in both MYCN high- and low-expression NB cells. Furthermore, knockdown of MYCN downregulated the expression of EWSR1 (Supplementary Figure 2). Thus, overexpression of EWSR1 or an unidentified *EWS* fusion partner, may be a previously unrecognized downstream effect of *MYCN* amplification in NB.

Taken together, in this study, we demonstrate for the first time that YK-4-279 is cytotoxic to various NB cells (both *MYCN* amplified and *MYCN* nonamplified) and can induce cell death *in vitro* and *in vivo*. In addition, it potentiated the anticancer effect of Dox. The data presented here suggest that YK-4-279 might be beneficial in treatment of NB and deserves further investigation and clinical validation.

## MATERIALS AND METHODS

### Cell lines and cell culture

Human NB cell lines including *MYCN* nonamplified (SK-N-AS, SH-SY5Y, LA-N-6, and CHLA-255), and *MYCN* amplified (NB-19, NGP, and IMR-32) cell lines (Table 2), were routinely cultured as described previously [36]. NB-19 cell line was kindly provided by Dr. A. Davidoff (St. Jude’s Children’s Research Hospital, Memphis, TN, USA), and LA-N-6 cell line was from Dr. R. Seeger (Children’s Hospital of Los Angeles, Los Angeles, CA, USA). The SH-SY5Y cell line with stable expression of luciferase was generated by transduction with LentiV2-luciferase expression virus. The stable cell line was established after five days of selection with puromycin at a concentration of 0.5 μg/ml.

**Table 2:** *MYCN* amplification in the human NB cell lines

Cell lines	*MYCN*
SK-N-AS	non-amplified
SH-SY5Y	non-amplified
CHLA-255	non-amplified
LA-N-6	non-amplified
NB-19	amplified
NGP	amplified
IMR-32	amplified

### Clinical patient cohorts

The Kocak dataset includes 649 [37] and the Versteeg dataset includes 88 primary NB tumor profiles and are publically available from the gene expression databases at the R2: Genomic Analysis and Visualization Platform (http://hgserver1.amc.nl/cgi-bin/r2/main.cgi).

### Antibodies and reagents

The anti-PARP (#9532), anti-Caspase 3 (#9662), anti-mouse (#7076) and anti-rabbit (#7074) antibodies were purchased from Cell Signaling Technology (Danvers, MA, USA). The antibody against EWS was purchased from Santa Cruz Biotechnology (#sc-1102, Dallas, TX, USA). YK-4-279 (#S7679) was purchased from Selleck Chemicals (Houston, TX, USA) and prepared according to the manufacturer’s recommendations. Dox (#D1515) and the anti-β-actin antibody (#A2228) were purchased from Sigma-Aldrich.

### Cytotoxicity assay and colony formation assay

Cytotoxicity was measured with Cell Counting Kit-8 (CCK-8) (Dojindo Laboratories, Rockville, MA, USA) following the manufacturer’s instructions. Briefly, NB cells seeded in 96-well plates at a density of 5 × 10^3^ cells per well were either allowed to grow in media alone or media containing increasing concentrations of YK-4-279, Dox, or their combinations. Seventy-two or 48 h later, cells were observed and photographed by the optical microscope, and then quantified with the CCK-8 assay. The absorbance of each well was measured at 450 nm and plotted for the cell viability curve. For the colony formation assay, cells seeded in 6-well plates at 5 × 10^3^ cells per well were incubated with YK-4-279 at 0 μM, 1 μM, or 3 μM for 72 h, then with drug-free medium for two weeks. After that, cells were fixed and stained with methanol/crystal violet and photographed. Each experiment was performed in triplicates.

### Immunoblotting

After each treatment, total proteins were extracted by lysing cells in RIPA buffer supplemented with protease inhibitor and phosphatase inhibitor cocktail as described previously [37]. The cell lysates were subjected to 10% or 15% SDS–PAGE electrophoresis and transferred to polyvinylidence fluoride (PVDF) membranes, followed by immunoblotting with primary antibodies, and horseradish peroxidase-conjugated antibodies against rabbit or mouse IgG. The membranes were developed using the ECL-Plus Western blotting system (GE Healthcare Biosciences Corp., Pittsburgh, PA, USA) according to the manufacturer’s instruction.

### Propidium iodide (PI) staining and fluorescence-activated cell sorting (FACS) assay

The experiment was performed as described previously [38]. Briefly, NB cells were seeded in 6 cm dishes and treated with 1 μM or 3 μM of YK-4-279 for 24 h. Cells were harvested by dissociation buffer, washed in ice-cold PBS and then stained with PI (#51-66211E; BD Biosciences, San Jose, CA, USA) and analyzed by FACS. Unstained cells were used as a negative control and untreated cells were used as a control for treated cells. Then analysis was performed on a LSR-II flow cytometer (BD Biosciences) using BD FACDiva software v. 6.0.

### Quantitative reverse transcription polymerase chain reaction (qRT-PCR) assay

EWSR1 mRNA expression in different NB cell lines was measured using qRT-PCR assay. Extraction of RNA, cDNA synthesis and qRT-PCR was performed as described previously [37]. Assays were performed in triplicates for each sample using Power SYBR Green PCR Master Mix (Applied Biosystems) and normalized to GAPDH. The primers were designed using Primer 3.0 software and are shown in Table 3.

**Table 3:** Primers for qRT-PCR

Gene name	Primer sequence
EWSR1	F: GGACGTTGAGAGAACGAGGA
	R: CAGTGGGCTGTCCATAGGTT
ACTB	F: ACCGCGAGAAGATGACCCAG
	R: TTAATGTCACGCACGATTTCCC

### Generation of stable NB cells expressing shRNA targeting EWS

A TRCB2 vector was used to generate shRNA plasmids for EWSR1. The following target sequences for EWSR1 have been selected shown in Table 4. The authenticity of these plasmids was confirmed by sequencing. The TRCB2-EWSR1 construct was transfected into HEK293T cells with Lentivirus packing vectors using PEI transfection reagent. Viral supernatants were collected after 48 h. NB cell lines (SK-N-AS, SH-SY5Y, NB-19, and NGP) were incubated with virus containing medium in the presence of 4 μg/ml polybrene (Sigma Aldrich). Stable cell lines were established after five days of puromycin (0.5 μg/ml) selection and knockdown of the target gene was confirmed by Western blot.

**Table 4:** siRNA sequence

Name	siRNA sequence
si-control	CTGGCATCGGTGTGGATGA
si-EWSR1-1	AAGAAGCCTCCAATGAACAGT
si-EWSR1-2	AACCGAGCAGCTATGGACAGC

### Orthotopic mouse model of NB

Five to six-week-old female athymic Ncr nude mice (Taconic Biosciences, Hudson, NY, USA) maintained under barrier conditions were used for xenograft study. Mice were implanted using an orthotopic xenograft model of NB as previously described [22, 23]. Briefly, 1 × 10^6^ human luciferase-transduced SH-SY5Y cells suspended in 0.1 ml of PBS were surgically implanted in the left renal capsule of the mice. Tumor growth was monitored weekly by bioluminescent imaging (IVIS Lumina XR System, Caliper Life Sciences, Hopkinton, MA, USA). After two weeks, mice bearing tumors with similar sizes were randomly divided into two groups: a DMSO control group and a YK-4-279 treatment group. Four mice in each group were treated for two weeks (YK-4-279 50 mg/kg or DMSO by intraperitoneal [i.p.] injection every other day). Two more sets of four mice each that had been bearing the SH-SY5Y tumors for four weeks were subsequently treated with either YK-4-279 or DMSO *via* i.p. injection for five days. At the end of the treatment, all mice were euthanized. Tumors and the right kidneys (control) were resected and weighed. Tumors were then submitted for Western blot analysis. All animal experiments are approved by Institutional Animal Care and Use Committee of Baylor College of Medicine.

### Statistical analysis

Statistical analysis was performed using GraphPad Prism 5 software. All values were presented as mean ± standard deviation (SD). * *P*<0.05, ** *P*<0.01, *** *P*<0.001 were considered to be statistically significant. Student’s *t* test (two-tailed) or *ANOVA* (Dunnett’s multiple comparison post-test) were used to analyze the difference between drug treatment groups and control groups. Survival analyses were performed using Kaplan-Meier method and two-sided log-rank tests.

## SUPPLEMENTARY MATERIALS FIGURES



## Abbreviations

NB: neuroblastoma
RHA: RNA helicase A
Dox: doxorubicin

## References

[1] Howlader NA, Krapcho M, Garshell J, Miller D, Altekruse SF, Kosary CL, Yu M, Ruhl J, Tatalovich Z, Mariotto A, Lewis DR, Chen HS, Feuer EJ (2015). SEER Cancer Statistics Review, 1975-2012.

[2] National Cancer Institute (2015). Neuroblastoma Treatment (PDQ): Health Professional Version. PDQ Cancer Information Summaries.

[3] Pinto NR, Applebaum MA, Volchenboum SL, Matthay KK, London WB, Ambros PF, Nakagawara A, Berthold F, Schleiermacher G, Park JR, Valteau-Couanet D, Pearson AD, Cohn SL (2015). Advances in risk classification and treatment strategies for neuroblastoma. J Clin Oncol.

[4] Brodeur GM, Iyer R, Croucher JL, Zhuang T, Higashi M, Kolla V (2014). Therapeutic targets for neuroblastomas. Expert opinion on therapeutic targets.

[5] Irwin MS, Park JR (2015). Neuroblastoma: paradigm for precision medicine. Pediatr Clin North Am.

[6] Barone G, Anderson J, Pearson AD, Petrie K, Chesler L (2013). New strategies in neuroblastoma: Therapeutic targeting of MYCN and ALK. Clin Cancer Res.

[7] Louis CU, Shohet JM (2015). Neuroblastoma: molecular pathogenesis and therapy. Annu Rev Med.

[8] Schleiermacher G, Janoueix-Lerosey I, Delattre O (2014). Recent insights into the biology of neuroblastoma. Int J Cancer.

[9] McArt DG, Dunne PD, Blayney JK, Salto-Tellez M, Van Schaeybroeck S, Hamilton PW, Zhang SD (2013). Connectivity mapping for candidate therapeutics identification using next generation sequencing RNA-seq data. PLoS One.

[10] McArt DG, Zhang SD (2011). Identification of candidate small-molecule therapeutics to cancer by gene-signature perturbation in connectivity mapping. PLoS One.

[11] Travelli C, Drago V, Maldi E, Kaludercic N, Galli U, Boldorini R, Di Lisa F, Tron GC, Canonico PL, Genazzani AA (2011). Reciprocal potentiation of the antitumoral activities of FK866, an inhibitor of nicotinamide phosphoribosyltransferase, and etoposide or cisplatin in neuroblastoma cells. The Journal of pharmacology and experimental therapeutics.

[12] Hanmod SS, Wang G, Edwards H, Buck SA, Ge Y, Taub JW, Wang Z (2015). Targeting histone deacetylases (HDACs) and Wee1 for treating high-risk neuroblastoma. Pediatric blood & cancer.

[13] Russell MR, Levin K, Rader J, Belcastro L, Li Y, Martinez D, Pawel B, Shumway SD, Maris JM, Cole KA (2013). Combination therapy targeting the Chk1 and Wee1 kinases shows therapeutic efficacy in neuroblastoma. Cancer research.

[14] Erkizan HV, Kong Y, Merchant M, Schlottmann S, Barber-Rotenberg JS, Yuan L, Abaan OD, Chou TH, Dakshanamurthy S, Brown ML, Uren A, Toretsky JA (2009). A small molecule blocking oncogenic protein EWS-FLI1 interaction with RNA helicase A inhibits growth of Ewing’s sarcoma. Nat Med.

[15] Awad O, Yustein JT, Shah P, Gul N, Katuri V, O’Neill A, Kong Y, Brown ML, Toretsky JA, Loeb DM (2010). High ALDH activity identifies chemotherapy-resistant Ewing’s sarcoma stem cells that retain sensitivity to EWS-FLI1 inhibition. PLoS One.

[16] Rahim S, Beauchamp EM, Kong Y, Brown ML, Toretsky JA, Uren A (2011). YK-4-279 inhibits ERG and ETV1 mediated prostate cancer cell invasion. PLoS One.

[17] Barber-Rotenberg JS, Selvanathan SP, Kong Y, Erkizan HV, Snyder TM, Hong SP, Kobs CL, South NL, Summer S, Monroe PJ, Chruszcz M, Dobrev V, Tosso PN (2012). Single enantiomer of YK-4-279 demonstrates specificity in targeting the oncogene EWS-FLI1. Oncotarget.

[18] Hong SH, Youbi SE, Hong SP, Kallakury B, Monroe P, Erkizan HV, Barber-Rotenberg JS, Houghton P, Uren A, Toretsky JA (2014). Pharmacokinetic modeling optimizes inhibition of the ’undruggable’ EWS-FLI1 transcription factor in Ewing Sarcoma. Oncotarget.

[19] Rahim S, Minas T, Hong SH, Justvig S, Celik H, Kont YS, Han J, Kallarakal AT, Kong Y, Rudek MA, Brown ML, Kallakury B, Toretsky JA (2014). A small molecule inhibitor of ETV1, YK-4-279, prevents prostate cancer growth and metastasis in a mouse xenograft model. PLoS One.

[20] van der Ent W, Jochemsen AG, Teunisse AF, Krens SF, Szuhai K, Spaink HP, Hogendoorn PC, Snaar-Jagalska BE (2014). Ewing sarcoma inhibition by disruption of EWSR1-FLI1 transcriptional activity and reactivation of p53. J Pathol.

[21] Minas TZ, Han J, Javaheri T, Hong SH, Schlederer M, Saygideger-Kont Y, Celik H, Mueller KM, Temel I, Ozdemirli M, Kovar H, Erkizan HV, Toretsky J (2015). YK-4-279 effectively antagonizes EWS-FLI1 induced leukemia in a transgenic mouse model. Oncotarget.

[22] Patterson DM, Shohet JM, Kim ES (2011). Preclinical models of pediatric solid tumors (neuroblastoma) and their use in drug discovery. Curr Protoc Pharmacol.

[23] Shi Y, Ma IT, Patel RH, Shang X, Chen Z, Zhao Y, Cheng J, Fan Y, Rojas Y, Barbieri E, Chen Z, Yu Y, Jin J (2015). NSC-87877 inhibits DUSP26 function in neuroblastoma resulting in p53-mediated apoptosis. Cell Death Dis.

[24] Zucman J, Melot T, Desmaze C, Ghysdael J, Plougastel B, Peter M, Zucker JM, Triche TJ, Sheer D, Turc-Carel C (1993). Combinatorial generation of variable fusion proteins in the Ewing family of tumours. EMBO J.

[25] Delattre O, Zucman J, Melot T, Garau XS, Zucker JM, Lenoir GM, Ambros PF, Sheer D, Turc-Carel C, Triche TJ (1994). The Ewing family of tumors--a subgroup of small-round-cell tumors defined by specific chimeric transcripts. N Engl J Med.

[26] Diede SJ (2014). Spontaneous regression of metastatic cancer: learning from neuroblastoma. Nat Rev Cancer.

[27] Sun W, Quan C, Huang Y, Ji W, Yu L, Li X, Zhang Y, Zheng Z, Zou H, Li Q, Xu P, Feng Y, Li L (2015). Constitutive ERK1/2 activation contributes to production of double minute chromosomes in tumour cells. J Pathol.

[28] Brodeur GM, Bagatell R (2014). Mechanisms of neuroblastoma regression. Nat Rev Clin Oncol.

[29] Shohet JM, Ghosh R, Coarfa C, Ludwig A, Benham AL, Chen Z, Patterson DM, Barbieri E, Mestdagh P, Sikorski DN, Milosavljevic A, Kim ES, Gunaratne PH (2011). A genome-wide search for promoters that respond to increased MYCN reveals both new oncogenic and tumor suppressor microRNAs associated with aggressive neuroblastoma. Cancer research.

[30] Kovar H (2005). Context matters: the hen or egg problem in Ewing’s sarcoma. Semin Cancer Biol.

[31] Kauer M, Ban J, Kofler R, Walker B, Davis S, Meltzer P, Kovar H (2009). A molecular function map of Ewing’s sarcoma. PLoS One.

[32] Lamhamedi-Cherradi SE, Menegaz BA, Ramamoorthy V, Aiyer RA, Maywald RL, Buford AS, Doolittle DK, Culotta KS, O’Dorisio JE, Ludwig JA (2015). An oral formulation of YK-4-279: preclinical efficacy and acquired resistance patterns in ewing sarcoma. Mol Cancer Ther.

[33] Selvanathan SP, Graham GT, Erkizan HV, Dirksen U, Natarajan TG, Dakic A, Yu S, Liu X, Paulsen MT, Ljungman ME, Wu CH, Lawlor ER, Uren A (2015). Oncogenic fusion protein EWS-FLI1 is a network hub that regulates alternative splicing. Proc Natl Acad Sci U S A.

[34] Burchill SA, Wheeldon J, Cullinane C, Lewis IJ (1997). EWS-FLI1 fusion transcripts identified in patients with typical neuroblastoma. Eur J Cancer.

[35] Brodeur GM, Seeger RC, Schwab M, Varmus HE, Bishop JM (1984). Amplification of N-myc in untreated human neuroblastomas correlates with advanced disease stage. Science.

[36] Zhang H, Dou J, Yu Y, Zhao Y, Fan Y, Cheng J, Xu X, Liu W, Guan S, Chen Z, Shi Y, Patel R, Vasudevan SA (2015). mTOR ATP-competitive inhibitor INK128 inhibits neuroblastoma growth via blocking mTORC signaling. Apoptosis.

[37] Sun W, Yu Y, Dotti G, Shen T, Tan X, Savoldo B, Pass AK, Chu M, Zhang D, Lu X, Fu S, Lin X, Yang J (2009). PPM1A and PPM1B act as IKKbeta phosphatases to terminate TNFalpha-induced IKKbeta-NF-kappaB activation. Cell Signal.

[38] Xu X, Hegazy WA, Guo L, Gao X, Courtney AN, Kurbanov S, Liu D, Tian G, Manuel ER, Diamond DJ, Hensel M, Metelitsa LS (2014). Effective cancer vaccine platform based on attenuated salmonella and a type III secretion system. Cancer research.

