# Supplementary material for: EWS-FLI1 and RNA helicase A interaction inhibitor YK-4-279 inhibits growth of neuroblastoma
Source: Oncotarget. 2017 Oct 19;8(55):94780–92. doi: 10.18632/oncotarget.21933 (PMC5706912; doi:10.18632/oncotarget.21933)
Supplement: Supplementary file 1 [file oncotarget-08-94780-s001.pdf]

EWS-FLI1 and RNA helicase A interaction inhibitor YK-4-279 inhibits growth of neuroblastoma

SUPPLEMENTARY MATERIALS

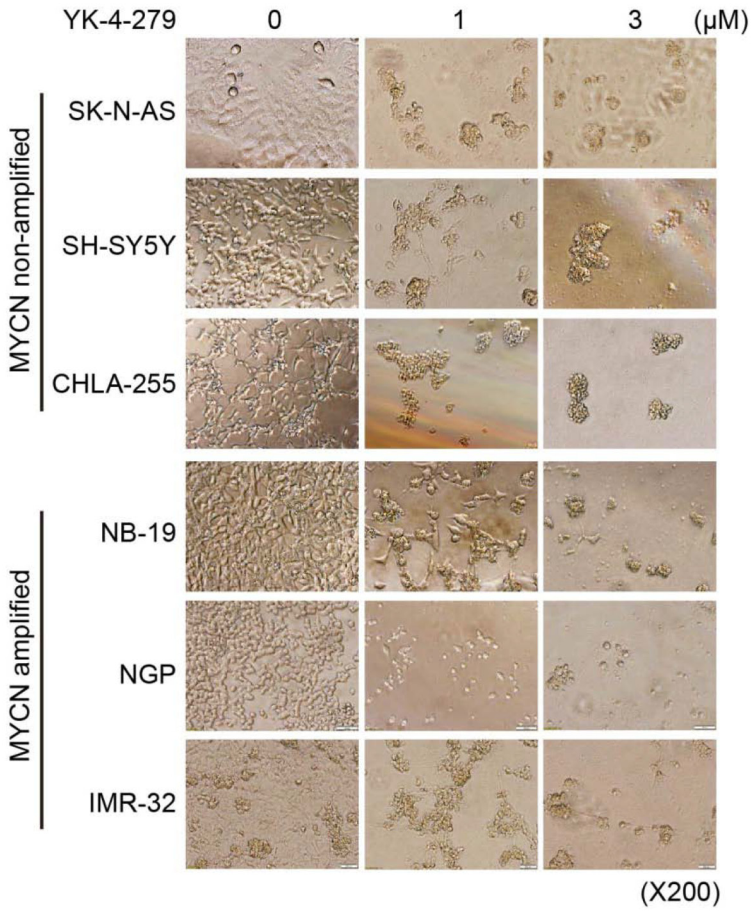

Supplementary Figure 1: Photographs of the NB cells treated with YK-4-279. Six human NB cell lines SK-N-AS, SH-SY5Y, CHLA-255, NB-19, NGP, and IMR-32 were treated with YK-4-279 at the concentrations of 0, 0.01 μM, 0.03 μM, 0.1 μM, 0.3 μM, 1 μM, 3 μM, 10 μM, 30 μM, and 100 μM for 72 h, and then photographed by the optical microscope.

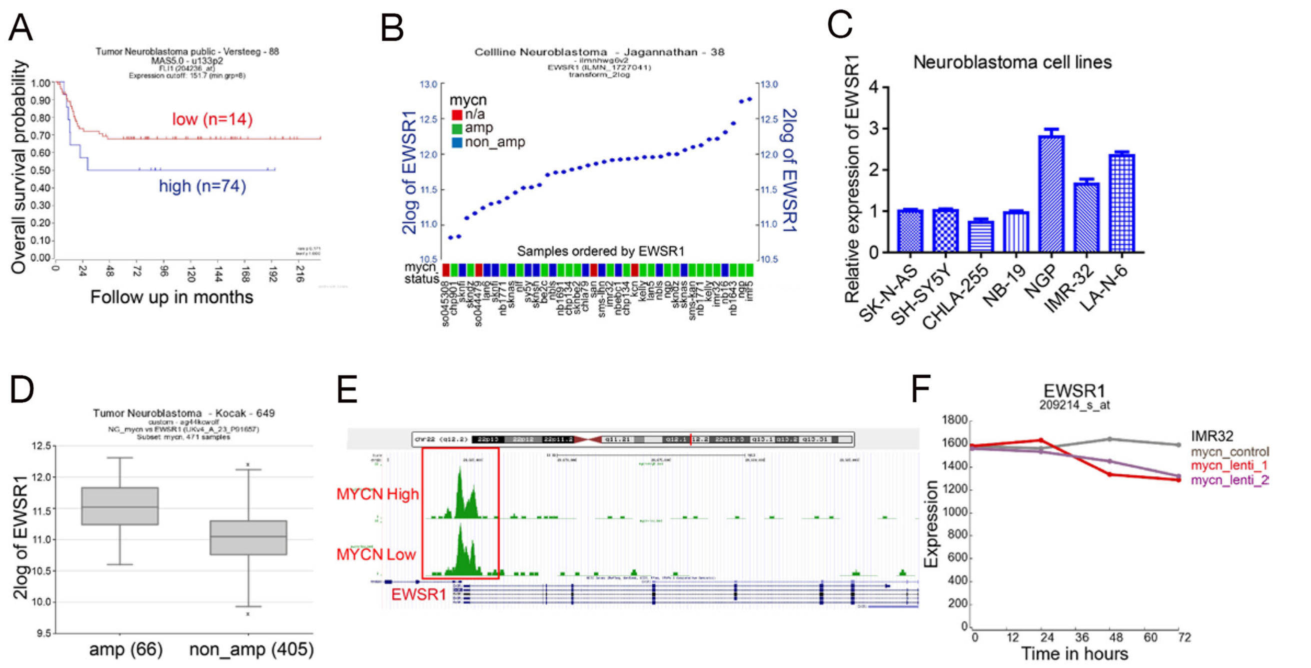

Supplementary Figure 2: (A) Kaplan-Meier curves show the probability of overall survival of 88 patients in the Versteeg dataset based on FLI1 expression level at the R2: Genomic Analysis and Visualization Platform. **(B)** The expression level of EWSR1 in 38 NB cell lines in Jagannathan dataset in R2. **(C)** The expression level of EWSR1 in seven NB cell lines, SK-N-AS, SH-SY5Y, CHLA-255, NB-19, NGP, IMR-32, and LA-N-6 by qRT-PCR analysis. **(D)** The expression level of EWSR1 in 649 NB samples with MYCN amplified and nonamplified in the Kocak dataset at the R2: Genomic Analysis and Visualization Platform. **(E)** MYCN binds to the 5'-UTR of EWSR1 in both MYCN high and low expression NB cells in MYCN-ChIP-seq data. **(F)** Knockdown MYCN by Lenti downregulate the expression of EWSR1 in NB cells in R2: Time Series dataset.
